# “SURVIVING AGING” - LOW-INCOME OLDER ADULTS AND THEIR PERSPECTIVES ON THE MEANING OF LIFE: A QUALITATIVE ANALYSIS

**DOI:** 10.1093/geroni/igae098.4275

**Published:** 2024-12-31

**Authors:** Daniel David, Kim Hadson, Abraham Brody

**Affiliations:** NYU College of Nursing, New York, New York, United States; NYU Rory Meyers College of Nursing, New York, New York, United States; New York University, New York, New York, United States

## Abstract

**Introduction:**

Low-income older adults often face challenging life circumstances, making it essential to explore how they derive meaning in life. This study examines the perspectives of residents in Medicaid-supported assisted living facilities on what gives their lives meaning, shedding light on their psychosocial needs and well-being.

**Methods:**

Qualitative interviews were conducted with 60 residents across three Medicaid-supported assisted living facilities in New York City. Participants responded to the question, “What gives your life meaning?” The sample was diverse, including individuals navigating aging, illness, and limited resources. Data were analyzed using content analysis to identify common themes.

**Results:**

Five key themes emerged: 1) Overcoming Challenges, 2) Seeking Meaningful Relationships with Family and Aides, 3) Focusing on and Accepting the Present, 4) Personal Growth and Community Contribution, and 5) Maintaining Autonomy. A subset expressed an existential view, stating, “There is no meaning.” Despite numerous challenges, most residents sought meaning late in life by focusing on strengths rather than barriers.

**Discussion:**

The findings highlight the complexity of meaning-making in later life, particularly among low-income older adults in under-resourced settings. While challenges are significant, the ability to find meaning through relationships, personal growth, and autonomy suggests critical avenues for intervention. Existential concerns raised by some participants indicate a need to address these perspectives within psychosocial support frameworks.

**Conclusion:**

This study offers insights into how low-income older adults in assisted living derive meaning. Emphasizing strengths over barriers underscores the importance of targeted interventions to support their psychosocial needs and enhance their quality of life.

